# Non-coding RNAs in Mesenchymal Stem Cell-Derived Extracellular Vesicles: Deciphering Regulatory Roles in Stem Cell Potency, Inflammatory Resolve, and Tissue Regeneration

**DOI:** 10.3389/fgene.2017.00161

**Published:** 2017-10-25

**Authors:** Farah Fatima, Karin Ekstrom, Irina Nazarenko, Marco Maugeri, Hadi Valadi, Andrew F. Hill, Giovanni Camussi, Muhammad Nawaz

**Affiliations:** ^1^Department of Rheumatology and Inflammation Research, Institute of Medicine, Sahlgrenska Academy, University of Gothenburg, Gothenburg, Sweden; ^2^Department of Pathology and Forensic Medicine, Ribeirão Preto Medical School, University of São Paulo, São Paulo, Brazil; ^3^Department of Biomaterials, Institute of Clinical Sciences, Sahlgrenska Academy, University of Gothenburg, Gothenburg, Sweden; ^4^Faculty of Medicine, Institute for Infection Prevention and Hospital Epidemiology, Medical Centre, University of Freiburg, Freiburg, Germany; ^5^Department of Biochemistry and Genetics, La Trobe Institute of Molecular Science, La Trobe University, Bundoora, VIC, Australia; ^6^Department of Molecular Biotechnology and Health Sciences, University of Turin, Torino, Italy

**Keywords:** extracellular vesicles, exosomes, mesenchymal stem cells, non-coding RNA, gene expression regulation, matrix remodeling, inflammatory resolve, tissue repair

## Abstract

Extracellular vesicles (EVs) are heterogeneous populations of nano- and micro-sized vesicles secreted by various cell types. There is mounting evidence that EVs have widespread roles in transporting proteins, lipids, and nucleic acids between cells and serve as mediators of intercellular communication. EVs secreted from stem cells could function as paracrine factors, and appear to mimic and recapitulate several features of their secreting cells. EV-mediated transport of regulatory RNAs provides a novel source of trans-regulation between cells. As such, stem cells have evolved unique forms of paracrine mechanisms for recapitulating their potencies with specialized functions by transporting non-coding RNAs (ncRNAs) via EVs. This includes the dissemination of stem cell-derived EV-ncRNAs and their regulatory effects elicited in differentiation, self-renewal, pluripotency, and the induction of reparative programs. Here, we summarize and discuss the therapeutic effects of mesenchymal stem cell-derived EV-ncRNAs in the induction of intrinsic regenerative programs elicited through regulating several mechanisms. Among them, most noticeable are the EV-mediated enrichment of ncRNAs at the injury sites contributing the regulation of matrix remodeling, epithelial mesenchymal transitions, and attraction of fibroblasts. Additionally, we emphasize EV-mediated transmission of anti-inflammatory RNAs from stem cells to injury site that potentially orchestrate the resolution of the inflammatory responses and immune alleviation to better facilitate healing processes. Collectively, this knowledge indicates a high value and potential of EV-mediated RNA-based therapeutic approaches in regenerative medicine.

## Introduction

Extracellular vesicles (EVs) comprise a heterogeneous population of nano- and micro-sized vesicles secreted by virtually all cell types studied so far (Yáñez-Mó et al., 2015; Mateescu et al., 2017). The best-described EVs are the exosomes and microvesicles, which differ in their respective sizes, shapes, and origins. Exosomes are produced through the endocytic pathway, followed by the fusion of multivesicular endosomes with the plasma membrane where they are then released into the extracellular space (for detailed mechanisms see Nawaz et al., 2014). During the process of their biogenesis, EVs acquire repertoire of bioactive cargo such as proteins and lipids (Keerthikumar et al., 2016), coding- and non-coding RNAs (ncRNAs) including both microRNAs and long noncoding RNAs (miRNAs, lncRNAs) (reviewed by Fatima and Nawaz, 2017c), and presumably DNA (Thakur et al., 2014). The secreted EVs serve as mediators of intercellular communication (Ratajczak et al., 2006b; Mathivanan et al., 2010; Nawaz and Fatima, 2017), could disseminate biological information between cells and contribute as paracrine factors in health and disease (Bellingham et al., 2012; Buzas et al., 2014; Hoshino et al., 2015). EVs not only exchange biological material between neighboring cells but can also travel long distances, allowing the dissemination of genetic content between distal organs and regulate gene expression of host tissues (Fatima and Nawaz, 2017a; Thomou et al., 2017).

A complete understanding of stem cell biology is a prerequisite for gaining mechanistic insights into human diseases. The current applications of stem cells in translational medicine take advantage of their potency for regeneration and repairing tissue damage. The best studied in this context are the mesenchymal stem cells (MSCs). According to the International Society for Cellular Therapy, MSCs are defined as plastic adherent cells with the capacity to differentiate into osteoblasts, chondrocytes, myocytes, and adipocytes (Dominici et al., 2006). MSCs express markers such as CD73, CD90, and CD105, and lack the expression of several markers including CD14, CD34, CD45, or CD11b, CD79-α, or CD19 and HLA-DR surface molecules (Dominici et al., 2006).

The commonly considered sources of MSCs are bone marrow (BM), adipose tissue, the umbilical cord, nervous tissue, dental pulp, amniotic fluid, the placenta, and menstrual blood (Hass et al., 2011; Eirin et al., 2014). MSCs derived from these sources represent remarkable differences in morphology, proliferation, self-renewal ability, and differentiation potential (Dominici et al., 2006). Interestingly, their capacity to differentiate toward osteoblasts, chondrocytes, myocytes, and adipocytes, coupled with their ability to stay activated during injury and colonization to injury site offer a promising source in tissue regeneration. Although, the differentiation potential of MSCs is considerably less than that of embryonic stem cells (ESCs) as well as from induced pluripotent stem cells (iPS), they nevertheless hold greater promise for cell-based clinical applications (Uccelli et al., 2008). The benefits of MSC-based therapies are evident from their success in ameliorating the symptoms of many diseases including, diabetes, osteoarthritis, spinal cord injury, myocardial injury, graft vs. host disease, and bone repair shown in many clinical and preclinical models (Wei et al., 2013). The emerging biology of stem cells suggests that colonizing activity at the injury site is not always required and stem cells can extend their therapeutic effects in part via secreted paracrine factors at the site of injury (Nagaishi et al., 2016).

Recent studies propose that at least a part of MSC effects are mediated by MCS-derived EVs (Deregibus et al., 2007; Lai et al., 2016). MSC-derived EVs were tested in human patients with therapy-refractory graft-vs.-host disease. In this small study, using MSC-derived EVs from bone marrow donors to treat these patients, it was concluded that the MSC EVs were safe as well as effective in treating the disease (Kordelas et al., 2014). This study partly confirmed the direct application of MSC-derived EVs as an effective immune-suppressive factor. However, such therapeutic effects need to be confirmed in more patients.

NcRNAs are non-protein coding RNAs, which represent part of the genome that does not encode genetic information into proteins. In principle, ncRNAs are broadly categorized into short ncRNAs and long ncRNAs (lncRNAs) or long intergenic ncRNA (lincRNA). Several ncRNA species exist within the genome such as Piwi-interacting RNAs (piRNAs), small nuclear, and nucleolar RNAs (snRNAs, snoRNAs), and short interfering RNAs (siRNAs) among others described elsewhere (Fatima and Nawaz, 2017c). It is well-established that about 90% of the genome sequence is actively transcribed, but the translated proportion is <2% of the whole genome, which has been considered as “junk DNA.” But, the Encyclopedia of DNA Elements (ENCODE) project has revealed that more than 90% of the human genome contains functional ncRNAs (ENCODE Project Consortium, 2004, 2007), and thus untranslated fraction of the genome is no longer considered to be entirely without function (Lee, 2012).

The frequently studied class of ncRNAs are the miRNAs, which are precisely regulated during developmental processes. It is estimated that miRNAs regulate ~30% of all protein-coding genes and are fundamental in shaping the global transcriptome of eukaryotes (Filipowicz et al., 2008; Grosshans and Filipowicz, 2008). The miRNAs are customarily known to regulate gene expression at post-transcriptional level governing several key cellular pathways related to development, differentiation and cellular fates (Pasquinelli and Ruvkun, 2002; Ambros, 2003, 2004; Ivey and Srivastava, 2010). The contribution of ncRNAs in regulating healing and repair process of stem cells is now well-accepted and is thought to be more sophisticated than earlier studies suggested (Ounzain et al., 2013; Ounzain and Pedrazzini, 2015; Zhou et al., 2016). There is evolving evidence implicating stem cell-derived EVs in the maintenance of stem cell characteristics such as self-renewal, differentiation, maturation and cell fate determination (reviewed in Nawaz et al., 2016b). However, the roles of EV-derived ncRNA are only recently beginning to be explored. An increasing body of evidence has clarified that EV-ncRNAs could serve as potential mediators of the extended paracrine effects of stem cells. Since ncRNAs are central to gene regulation and cellular fates, it can be speculated that most of the EV-mediated regulatory roles elicited in cells/organs are mediated through ncRNAs. The ncRNAs are expressed in a tissue-specific manner, precisely regulated and actively involved in variety of developmental processes (Pasquinelli and Ruvkun, 2002; Ambros, 2003; Carrington and Ambros, 2003; Marson et al., 2008; Gangaraju and Lin, 2009; Pauli et al., 2011; Fatica and Bozzoni, 2014; Perry and Ulitsky, 2016). Lineage specific commitments of stem cells and the maintenance of their characteristic features such as pluripotency, self-renewal, differentiation, and efficiency of cellular reprogramming are largely regulated by ncRNAs (Dinger et al., 2008; Judson et al., 2009; Loewer et al., 2010; Shenoy and Blelloch, 2014; Xu et al., 2016; Deng et al., 2017; Hou et al., 2017; Wei et al., 2017; Zhang W. et al., 2017). Thus, the ncRNAs may govern the equilibrium between pluripotency and differentiation in the embryo and embryonic stem cells, and lineage specific fate decisions (Ivey and Srivastava, 2010; Flynn and Chang, 2014). Recent studies have shown that MSC-derived EVs are enriched in distinct ncRNA species such as miRNAs, tRNA, and Piwi-interacting RNA (piRNAs) which contribute to maintaining stem cell potency (Baglio et al., 2015), induction of cell survival and inhibition of cell differentiation of cord blood hematopoietic stem cells (De Luca et al., 2016). The comparison of transcriptomic (RNA-Seq) and proteomic profiles of ESC-derived EVs and EVs from human bone-marrow (BM-MSC) revealed distinctly different RNA profiles between EVs of two stem cell populations (Billing et al., 2016).

## Stem cell-derived ncRNAs and EVs: allies in stem cell potency

Evidence now exists to support that EVs mimic several features of their parent cells and profoundly contribute to stem cell fate decisions (Nawaz et al., 2016b). Ratajczak and colleagues provided the first evidence that stem cell-derived EVs contain mRNA transcripts for pluripotent transcriptional factors such as HoxB4, Nanog, Oct-4, and Rex-1, which can be horizontally transferred to recipient cells, supporting hematopoietic progenitor cells expansion (Ratajczak et al., 2006a). EV-mediated transfer of miRNAs downregulate vascular cell adhesion molecule (VCAM1) expression, contributing to hematopoietic progenitor cell mobilization (Salvucci et al., 2012).

Quesenberry and colleagues proposed that EV-mediated communication and exchange of genetic material is the continuum model of stem cell biology, where the differentiation decision of stem cells is conditioned by the cell cycle transit and environmental stimuli (Quesenberry et al., 2010). It is tempting to speculate that the dependency of progenitor cell differentiation and lineage commitment could be reprogrammed by continuous flow of genetic material bidirectionally between progenitors and differentiated cells (Nawaz et al., 2016b). Stem cells preferably keep population equilibrium between progenitors and the differentiated mature cells. Thus, a deficiency of mature cells in a particular tissue could be sensed by progenitors, which produce more progenies to be differentiated into mature cells. As such, this equilibrium could be facilitated by EV-mediated bidirectional exchange of genetic material, which favors stem cell populations to maintain a stable co-existence (Nawaz et al., 2016b).

**Figure 1:**
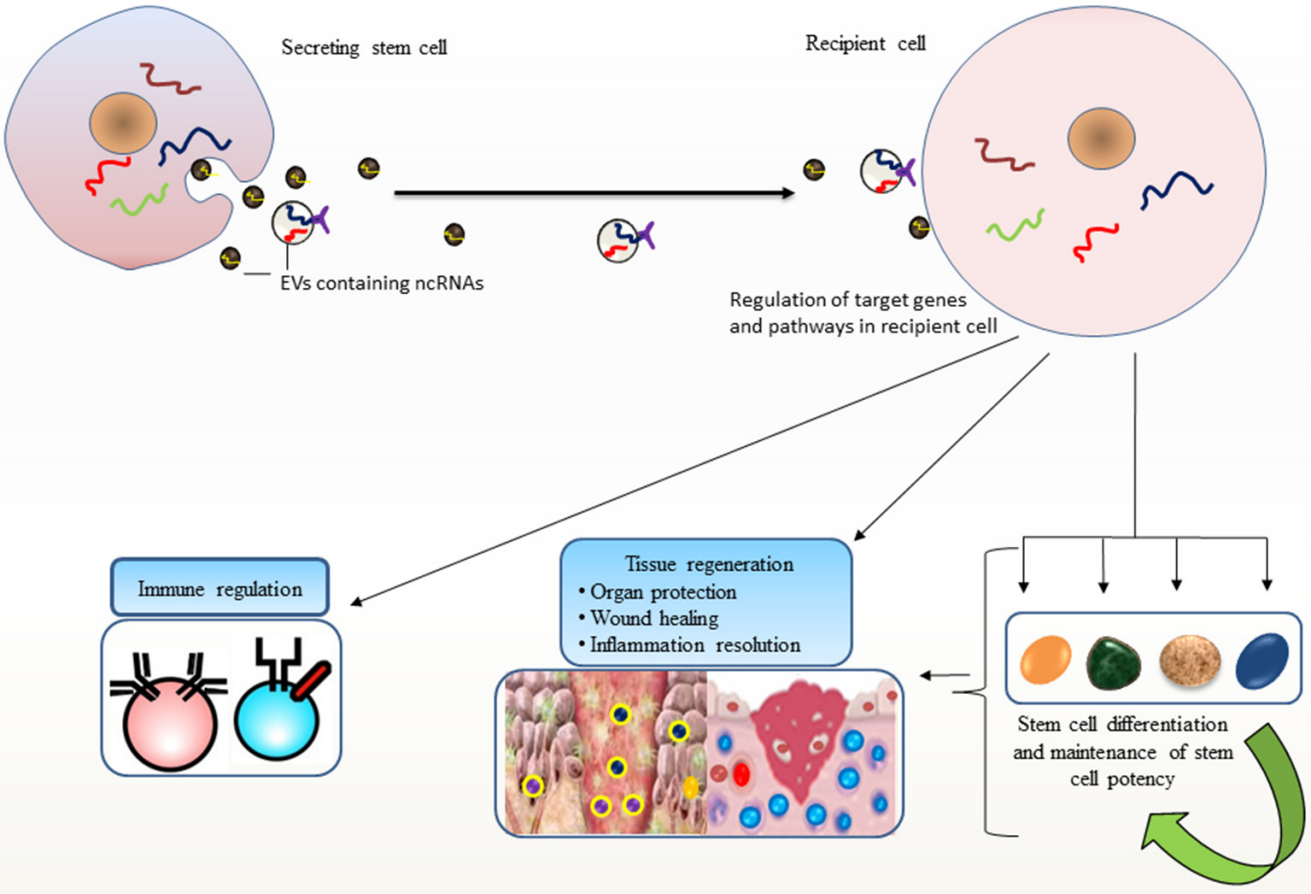
Stem cell potency and differentiation: Stem cells secrete extracellular vesicles (EVs) carrying non-coding RNAs (ncRNAs) that are transported to other cells. Such horizontal transfer is implicated in recapitulating variety of stem cell features in recipient cells, such as pluripotency, differentiation, and stem cell maintenance and their ability to facilitate regenerative processes. EV-mediated transport of ncRNAs elicits regulatory programs in recipient cells; maintain tissue homeostasis and immune regulation that may favor repair processes.

The secretion of a selective pattern of miRNAs from stem cells and their transfer to target cells via EVs raises enormous potential for stem cells to recapitulate lineage specific characteristics (Collino et al., 2010; Guo et al., 2011). Additional roles for EV-miRNAs in differentiation are observed where EV-miR-486 delivery confers a rapid response to hypoxia in erythroleukemia cells by targeting *Sirt1* gene, and modulates hypoxia-induced erythroid differentiation (Shi et al., 2017). Likewise, ESC-derived EVs could transport selective subset of miRNA and transcriptional factor related mRNAs which may induce pluripotency in their target cells and turn on early retinogenic program of differentiation (Katsman et al., 2012). It is increasingly being recognized that stem cells have evolved mechanisms for maintaining stem cell specific features at least, in part through EV-mediated dissemination of ncRNAs (Figure 1).

## Tissue regeneration and organ protection

The secretion of EVs from biologically active cells may be context dependent i.e., relating to disease progression or induction of regenerative programs (Fatima and Nawaz, 2015). As such, EV-mediated transportation of stem cell-derived ncRNA to the injured sites is considered one of the versatile regulatory routes of tissue regeneration and organ protection. This section will discuss roles of EVs in mediation of paracrine effects and the mechanisms in the context of tissue remodeling and repairing injuries.

### Matrix remodeling and inhibition of epithelial-mesenchymal-transition

MSC-derived EVs are demonstrated to optimize the matrix elements by activation of collagen regulation synthesis by stromal fibroblasts, which further support the healing processes (Zhang et al., 2015; Hu et al., 2016). MSCs transfer miR-125a to endothelial cells via EVs, which promotes the formation of endothelial tip cells and angiogenesis by repressing angiogenic inhibitor delta-like 4 (DLL4; Liang et al., 2016). Additionally, MSC-derived EVs containing miRNAs could inhibit the TGF-β/SMAD2 pathway and suppress myofibroblast differentiation during wound healing (Fang et al., 2016). The wound healing process is mainly facilitated by endothelial cell proliferation and fibroblast activation for which growth factors play a central role. Notably, the platelet-rich plasma (PRP) is rich source of growth factors and has a widespread role in repairing chronic wounds mainly through endothelial cell activation and angiogenesis. The role of PRP-derived EVs bearing the cargo of growth factors is much appreciated for the induction of fibroblast and endothelial cell proliferation and migration which favor angiogenesis and re-epithelialization in chronic wounds (Guo et al., 2017).

While the proliferation of fibroblasts facilitates matrix remodeling in favor of tissue repair, the excess number of fibroblasts may cause the thickening of the tissue and hinder the repair process. Epithelial-mesenchymal-transition (EMT) holds a central role in fibroblast functionality. In fact, EMT promotes the genesis of fibroblasts where the excess of fibroblasts may exhibit the phenomenon of organ fibrosis with deleterious effects in adult tissues (Kalluri and Neilson, 2003). Therefore, fibroblast optimization is essential for repairing defects, whereby inhibition of EMT potentially supports tissue repair (Câmara and Jarai, 2010; Xi et al., 2014). Recent studies show that MSC-derived EVs influence the inhibition of EMT during injuries in order to favor the healing process. In two concordant studies it was shown that the proximal tubular epithelial cells (PTEC) treated with TGF-β1 may repress E-cadherin and exhibit EMT associated morphological changes, whereas the cells administered with MSC-derived EVs may reverse the morphological changes by resuming the E-cadherin expression; allowing the protection of mice against renal failure (He et al., 2015; Wang et al., 2015a).

Notably, EVs from BM-MSCs demonstrate inhibitory effects on TGF-β1-mediated EMT in renal PTEC cells *in-vivo* (Wang et al., 2015a). This is interesting to consider that EMT inhibition was stronger in younger rats than older rats, indicating that renal protection is more active during young age and less in older age, and may play a role in the fibrosis of aging renal tissues (Wang et al., 2015a). In principle, such roles of EVs are projected by transferring selective patterns of miRNAs from MSCs to the injured renal PTEC cells, which inhibit EMT (He et al., 2015; Wang et al., 2015a). As stated earlier, EMT inhibition is potential choice for supporting tissue regenerative reprograms; EVs may serve new vehicle for inhibition of EMT and might be a useful therapeutic strategy in regenerative processes.

### Transcriptional repression of apoptotic genes: role of anti-apoptotic miRNAs

The contribution of stem cell-derived EVs is increasingly being recognized in ameliorating organ functions through preventing cell death, promoting cell survival, and progenitor's self-renewal. MSC-derived EVs transfer anti-apoptotic miRNAs to injured cells, which not only promote cell survival but may also elicit transcriptional modulation of target genes in injured renal epithelial cells as noticed in *in-vitro* as wells *in-vivo* models of kidney injury (Lindoso et al., 2014; Cantaluppi et al., 2015). More evidence describe that the MSC-derived EVs could transfer anti-apoptotic miRNAs to cardiomyocytes, which transcriptionally repress apoptotic genes in cardiomyocytes and facilitate cell survival with enhanced angiogenesis, ultimately ameliorating the cardiomyocytes functions (Yu et al., 2013; Wang et al., 2015b, 2017). Similarly, EVs from cardiac progenitor cells (CPCs) transport anti-apoptotic miRNAs, which target apoptotic genes and inhibit apoptosis in cardiomyocytes, stimulate tube formation in endothelial cells, which favor angiogenesis, and thus improve heart function (Barile et al., 2014; Xiao et al., 2016). Agarwal and colleagues reported miRNA-mediated reparative potential of CPC-derived EVs from pediatric patients (Agarwal et al., 2017). This study demonstrates that the functional improvements are associated with increased angiogenesis, reduced fibrosis, and improved hypertrophy, resulting in improved cardiac function and such outcomes are linked to miRNA functions. Shao et al. (2017) recently reported that MSCs and MSC-derived EVs exhibit similar miRNA expression profile, which could be one of the reasons that MSC-derived EVs can replace MSCs for cardiac repair. This indicates that that MSC-derived EVs could be used alone to promote cardiac repair and are superior to MSCs in repairing injured myocardium. Wang and colleagues argue that endometrium MSCs (EnMSCs) confer superior cardioprotection as compared to BM-MSCs or adipose tissue derived MSCs (AD-MSCs) showing miR-21 as a potential mediator of EnMSC therapy (Wang et al., 2017).

Recently a study has demonstrated that EVs secreted from cardiosphere-derived cells are highly enriched in Y RNA fragment (EV-YF1) (Cambier et al., 2017). EVs transfer YF1 to macrophages (YF1 transfection of macrophages) thereby inducing the *IL-10* transcription and secretion of IL-10 which ameliorates cardiomyocyte functions. Interestingly, EV-YF1-primed macrophages co-cultured with rat cardiomyocytes were potently protective against oxidatively stressed cardiomyocytes through induction of IL-10 expression. Additionally, the *in-vivo* intracoronary injection of EV-YF1 following ischemia/reperfusion reduced the infarct size (Cambier et al., 2017). Interestingly, a profound regenerative potential of cardiac stem cells (CSCs)-derived EVs has been demonstrated in a mice model of cardiomyopathy (Vandergriff et al., 2015). This study demonstrates the double advantage of EVs; firstly, the mice used in experiment was immunocompetent, but showed no adverse immune reaction against therapeutic EVs. Secondly, although mice received heterologous source of CSC-derived EVs (i.e., from human CSCs) but no cross reactivity was observed between EVs of two different sources.

### Cellular differentiation, reprogramming, and induction of repair programs

EVs derived from cells relay the stem cell messages to induce regeneration, resistance to apoptosis, and the induction of intrinsic repair cascades of injured cells. It has been shown that EVs secreted by MSCs undergoing osteogenic differentiation differs in the content of miRNAs compared to undifferentiated MSCs (Xu et al., 2014). These EVs are osteoinductive and are involved in RNA surveillance pathway, Wnt signaling, and RNA transport contributing to regulation of osteogenic differentiation (Xu et al., 2014). EVs carrying miR-196a from BM-MSCs govern the expression of osteogenic genes during osteoblasts differentiation (Qin et al., 2016). Additionally, EVs mediate a dialogue between osteoblasts and adipocytes during adipocyte and osteoblast differentiation. It has recently been suggested that the adipocyte/osteoblast balance is profoundly regulated at transcriptional level aided by EV-mediated transmission of miRNAs (Martin et al., 2015). EVs derived from miR-140-5p overexpressing human synovial MSCs could enhance cartilage tissue regeneration and prevent osteoarthritis of the knee in a rat model by regulating Wnt signaling and by blocking the side effects of ECM secretion (Tao et al., 2017b).

EV-mediated transfer of miRNAs to CPCs could induce glycolytic switch within CPCs, which supports adaptation to hypoxic stress in cardiovascular tissues (Ong et al., 2014). Interestingly, EVs released from glucose-treated ECs contained significantly lower amounts of miR-126 and elicited reduced endothelial repair capacity *in-vitro* and *in-vivo*. This indicates that under diabetic or hyperglycemic conditions EVs may exhibit poor capacity to vascular endothelial repair. Moreover, the expression analysis of miR-126 in circulating EVs from patients with stable coronary artery disease with and without diabetes mellitus revealed significantly reduced miR-126 expression in EVs from diabetic patients (Jansen et al., 2013). In fact, EVs released from apoptotic endothelial cells (ECs) transfer miR-126 and influence the repair of recipient human coronary artery ECs (Jansen et al., 2013). Endothelial progenitor cells have also been shown to exhibit cellular reprograming by transferring EV-miRNAs to renal cells which protect kidney against ischemia reperfusion injury (Cantaluppi et al., 2012). EVs from pericardial fluid (secreted from heart) could mediate vascular repair responses in ECs by transferring pericardial fluid-miRNAs and inhibiting *TGF-BR1* (Beltrami et al., 2017). This improves the survival, proliferation, and networking of ECs and could restore the angiogenic capacity of ECs, which promotes post-ischemic blood flow recovery *in-vivo* (Beltrami et al., 2017).

A recent study demonstrates that aging in mice initiates with a substantial loss of hypothalamic stem/progenitor cells (Zhang Y. et al., 2017). Conversely, aging retardation and lifespan extension were achieved in mid-aged mice that were locally implanted with healthy hypothalamic stem cells. This effect (i.e., slowing of aging) is mediated by stem cell-derived exosomal miRNAs in the cerebrospinal fluid and concomitant regulation of aging factors in the brain microenvironment (Zhang Y. et al., 2017). Additionally, EV-miRNAs from MSCs are transferred to neural cells where they could regulate nerve growth and exhibit neuro-protective effects in *in-vivo* animal models (Xin et al., 2012; Cui et al., 2016). Stem cell-derived EVs have also been implicated in the protection of eye functions during/and after laser injuries. In fact, MSC-derived EVs ameliorate retinal laser injury partially by downregulating monocyte chemotactic protein in the retina (Yu et al., 2016). Additionally, MSC-derived EVs transfer miRNAs and promote the survival of retinal ganglion cells and regeneration of axons in an *in-vivo* rat model (Mead and Tomarev, 2017). This suggests that MSC-derived EVs could be used as a tool for cell-free therapy for traumatic and degenerative ocular disease. EVs secreted from AD-MSCs contain MALAT1-ncRNA which promotes neural regeneration and enhanced neuronal survival through regulating PKCδII splicing (El Bassit et al., 2016). miR-181-5p-modified AD-MSCs are selectively transferred to damaged liver cells via EVs, and prevent liver fibrosis by activating autophagy and down-regulating Stat3, Bcl-2, fibronectin, collagen I, vimentin, and α-SMA in the hepatic cells (Qu et al., 2017). Several other EV-miRNAs, which are potential therapeutic source to organ protection and amelioration of injuries, are listed in Table 1.

**Table 1:** List of extracellular vesicle-microRNAs form stem cells and their roles in regulation of stem cell maintenance and healing processes.

**microRNAs**	**Stem cell source**	**Implications in tissue repair**	**References**
miR-148a, miR-532-5p, miR-378, let-7f	Porcine adipose-tissue derived MSCs	Modulation of angiogenesis, differentiation and adipogenesis	Eirin et al., 2014
miR-223, miR-564, miR-451, miR-142-3p	Human bone marrow derived MSCs and liver resident stem cells	Multi-organ development, cell survival and differentiation, immune system regulation	Collino et al., 2010
miR-486	CD34+ human hematopoietic cells (Erythroleukemia cells)	Regulation of erythroid differentiation	Shi et al., 2017
miRNAs of 290 cluster	Mouse embryonic stem cells	De-differentiation and regulation of pluripotency in Müller cells, induction of early retinogenic program of differentiation	Katsman et al., 2012
miR-199b, miR-218, miR-148a, miR-135b, miR-221	Human bone marrow-derived MSCs	Osteogenic differentiation	Xu et al., 2014
miR-196a	Human bone marrow-derived MSCs	Osteoblasts differentiation and expression of osteogenic genes	Qin et al., 2016
miR-140-5p	Human synovial MSCs	Cartilage tissue regeneration inhibition of osteoarthritis	Tao et al., 2017b
miR-21, -23a, -125b, and -145	Umbilical cord-derived MSCs	Cutaneous wound healing	Fang et al., 2016
miR-344a, miR-133b-3p, miR-294, miR-423-3p, miR-872-3p	Rat bone marrow-derived MSCs	Renal protection by reduced renal fibrosis and inhibition of EMT in aging kidney	Wang et al., 2015a
miR-148b-3p, 451, 485-3p, 495, 548c-3p, let-7a, 375, 410, 548c-5p, 561, 886-3p	Human bone marrow-derived MSCs	Renal protection by inhibiting apoptosis, enhanced cell survival, cytoskeleton reorganization, and recovery of process in PTECs	Lindoso et al., 2014
miR-221	Rat bone marrow MSC MSCs	Cardio-protection by enhanced cell survival, and inhibition of cardiomyocyte apoptosis	Yu et al., 2013
miR-21, miR-210	Mouse cardiac fibroblast-derived iPS	Cardio-protection by inhibiting cardiomyocyte apoptosis	Wang et al., 2015b
miR-21	Rat endometrium, bone marrow, and adipose tissues-derived MSCs	Cardiac protection through anti-apoptotic activity, enhanced cell survival, enhanced microvessel density and angiogenic effects	Wang et al., 2017
miR-21	Mice cardiac progenitor cells (CPCs)	Myocardium protection through anti-apoptotic activity, enhanced cell survival, cardiac repair	Xiao et al., 2016
miR-210, miR-132, miR-146a-3p	CPCs from atrial appendage explants from patients who underwent heart valve surgery	Cardiac protection through anti-apoptotic activity, tube formation and enhanced angiogenesis	Barile et al., 2014
miR-126, miR-210	Mice cardiac progenitor cells	Induction of glycolytic switch, activation of prosurvival kinases, cardiac protection	Ong et al., 2014
miR-145	Rat bone marrow-derived MSCs	Neuro-restoration through increased vascular and white matter remodeling	Cui et al., 2016
miR-133b	Rat bone marrow-derived MSCs	Neuro-protection through nerve growth and development	Xin et al., 2012
Let-7b, miR-21, miR-146a, and miR-181, miR-181c	Human umbilical cord-derived MSCs	Resolution of chronic inflammation and wound healing	Ti et al., 2015, 2016; Li et al., 2016
miR-146a	Human umbilical cord-derived MSCs	Inflammation regulation, macrophage M2 polarization and enhanced survival in sepsis mice	Song et al., 2017
miR-126-3p	Rat synovium MSCs	Cutaneous wound healing	Tao et al., 2017a
miR-290-295 cluster	Mouse embryonic stem cells	Cardiac protection through enhanced neovascularization, cardiomyocyte production and survival, and reduced fibrosis	Khan et al., 2015
miR-125b	Human chorionic plate-derived MSCs	Reduced fibrosis, and enhanced regeneration in damaged mice liver	Hyun et al., 2015
miR-223	Bone marrow-derived MSCs	Cardio-protection by protecting apoptosis and inflammatory response in sepsis mice model	Wang X. et al., 2015

*MSCs, Mesenchymal stem cells; iPS, induced pluripotent stem cells; CPCs, cardiac progenitor cells; PTECs, renal proximal tubular epithelial cells*.

## NcRNA-mediated inflammation regulation and healing

The successful healing process would require the alleviation of inflammatory insults during the course of tissue injury (White and Mantovani, 2013; Koniusz et al., 2016). In fact, the pro-inflammatory environment could modify the composition of EVs and the biological activities of immune effector cells; therefore, tissue regeneration therapy requires alleviation of inflammatory responses and immunosuppressive environment at the site of injury (Fatima and Nawaz, 2017b). This is possible through EV-assisted transfer/attraction of stem cell-derived ncRNAs at injury site, which efficiently govern inflammatory pathways.

The role of EVs have been well demonstrated in regulating inflammation resolution and immune regulation (Alexander et al., 2015; Nawaz et al., 2016a; Robbins et al., 2016; Silva et al., 2017), however, the contribution of EVs through ncRNAs in regulating inflammation resolution in the context of tissue repair is only more recently beginning to be explored. For instance, EVs carrying let-7b from preconditioned MSCs are shown to have avid effects on the regulation of macrophage plasticity and transition from inflammatory phase toward the proliferative phase (macrophage polarization), which favors the resolution of chronic inflammation (Ti et al., 2015). The umbilical cord MSC-derived EVs containing miR-21, miR-146a, and miR-181 have been demonstrated to regulate inflammation during the course of tissue repair (Ti et al., 2016).

Pretreatment with pro-inflammatory cytokines could improve the immunomodulatory efficacy of MSCs. Recent data suggest that the inflammatory effects of cytokines are regulated by MSC-derived EVs carrying miRNAs. For instance, IL-1β pre-treated MSCs (βMSCs) are shown to demonstrate upregulation of anti-inflammatory miRNAs such as miR-146a in response to IL-1β stimulation. In fact, IL-1β is transferred to macrophages via EVs and results in M2 polarization (characterized by anti-inflammatory phase), and increased survival in a septic mice model (Song et al., 2017). This is somewhat concordant with a study where bone marrow-derived macrophages efficiently take up EVs, which confer their switch from M1 to M2 phenotype enabling them to exhibit anti-inflammatory properties (Lo Sicco et al., 2017). In contrast, the inhibition of miR-146a may partially negate the immunomodulatory properties of βMSC-EVs. This indicates that IL-1β pre-treatment of MSCs could effectively enhance the immunomodulatory properties of MSCs through EV-mediated transfer of miR-146a. The educated βMSCs-EVs may contribute to enhanced immunomodulatory properties of βMSCs both *in-vitro* and *in-vivo* and may extend improved therapeutic application of MSCs in inflammatory disorders (Song et al., 2017).

**Figure 2:**
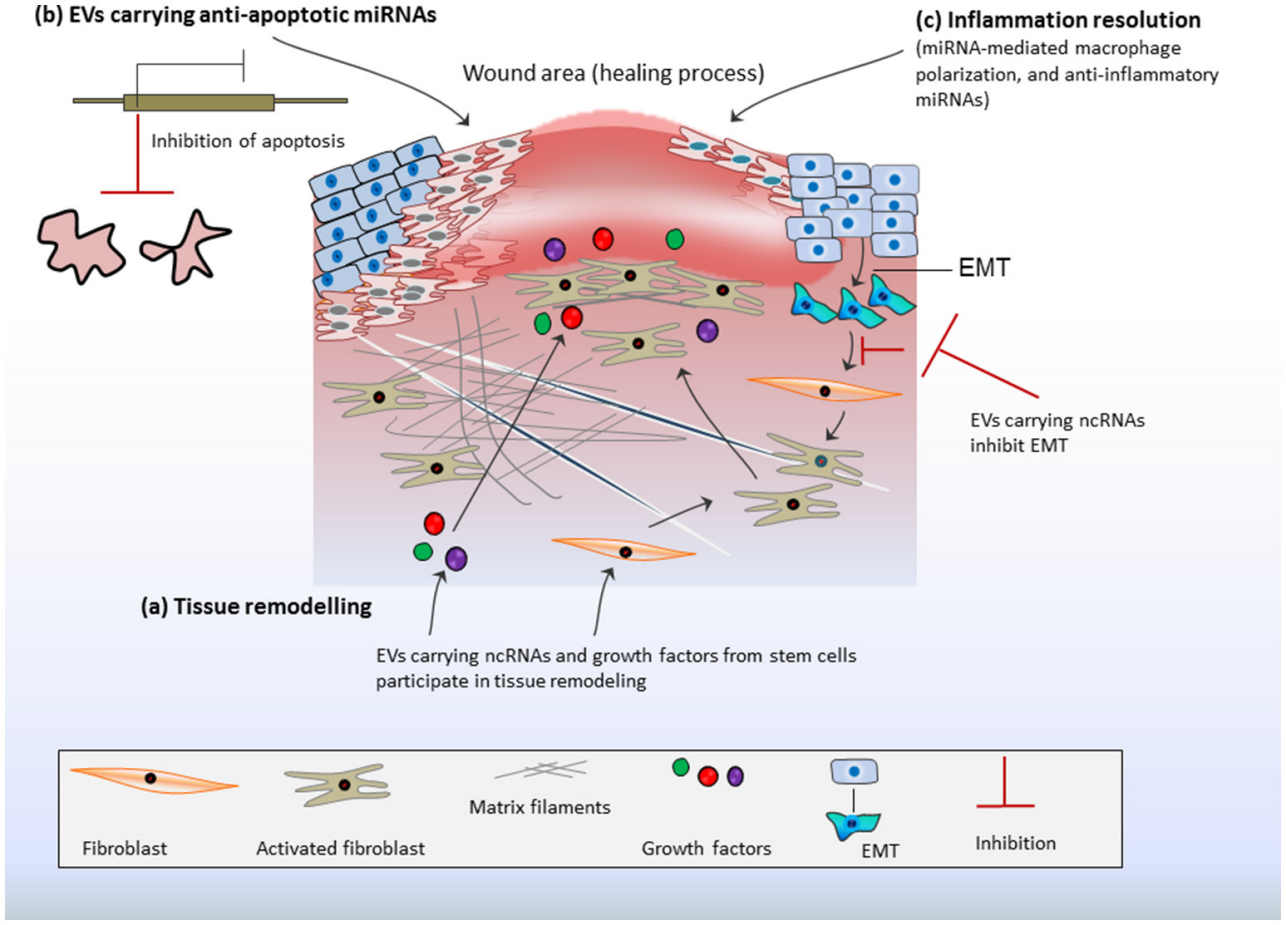
Stem cell-derived EV-ncRNAs and tissue repair: **(A)** Extracellular vesicles (EVs) carrying non-coding RNAs (ncRNAs) and growth factors may activate fibroblasts or/and endothelial cells. This promotes matrix reorganization and rapid responses in tissue regeneration. Fibroblasts proliferation is further enhanced by epithelial mesenchymal transition (EMT). This may contribute excess of fibroblasts at injury site or the formation of excess fibrous connective tissue. EV-ncRNAs regulate/inhibit EMT and ensure fibroblast optimization, which favors the repair process. **(B)** EVs transport anti-apoptotic miRNAs at the site of injury, which transcriptionally repress the expression of apoptotic genes and inhibit apoptosis thereby promoting cell survival during healing process. **(C)** EV-ncRNAs resolve inflammation by inducing macrophage polarization and transition from inflammatory phase to proliferative phase, whereas anti-inflammatory miRNAs from MSCs foster anti-inflammatory actions.

Epithelial lining is thought to serve a direct exposure to macrophages and inflammatory responses. Therefore, EVs from stimulated epithelial cells could promote macrophage activation *in-vitro* and facilitate the re-colonization of immunomodulatory cells *in-vivo* as noticed in bronchoalveolar lavage fluid (Lee et al., 2016). The macrophage-mediated pro-inflammatory effects are reliant on delivery of proinflammatory miRNAs from epithelial cells, indicating that the epithelial cell-EV-miRNAs are potential stimulators of macrophage-regulated lung inflammatory responses (Lee et al., 2016). Recently, *in-vivo* assays have demonstrated the profound effects of MSC-derived EVs in inflammation resolution favored toward enhanced diabetic cutaneous wound healing (Tao et al., 2017a). MiR-181c expression in human umbilical cord MSC-derived EVs could reduce burn-induced inflammation by downregulating the Toll-like receptor 4 (TLR4) signaling pathway (Li et al., 2016). Interestingly, EVs from human iPS could be engineered for siRNA delivery to human primary pulmonary microvascular endothelial cells that alleviate inflammatory responses in recipient cells by selective gene silencing of inflammatory genes (Ju et al., 2017). Collectively, such features of EVs makes them potential tools for immune/inflammatory resolve that is prerequisite for repairing processes (Figure 2).

## Concluding remarks

Since ncRNAs are expressed endogenously and regulate several cellular process through orchestrating gene expression organization of cells in *cis*; the secretion of ncRNAs via EVs could be envisaged for the purpose that cells might have evolved mechanism of trans-regulation between cells (Fatima and Nawaz, 2017c). EV-assisted transportation of ncRNAs between long distance organs is a newly recognized mechanism of gene expression regulation and extending the physiological and pathological communications between organs (Thomou et al., 2017). In this context, stem cells may secrete ncRNAs via EVs and deliver them to injured sites for inducing and regulating tissues' intrinsic programs. The precise knowledge of such mechanisms could help developing strategies for engineering EVs with therapeutic RNAs and delivering them to injured sites. Keeping in view the proposition that ncRNAs exhibit heterogeneous regulatory mechanisms; it would be essential to determine the functional readouts arising from ncRNA regulatory effects in injured tissues.

Despite improvements in the approaches applied to tissue repair and organ transplantation over the last decade, cell-based therapies still have potential risks such as, the increased risk of infection, toxicity, tumorigenicity, and immunogenicity. Moreover, cell-based therapies need to consider additional complications such as off-target effects after transplant such as genetic instability, loss of functional properties or induction of senescence, immune-mediated rejection (graft vs. host disease), and the transformation of resident cells into malignant phenotypes, which collectively could limit the therapeutic applications of stem cells. More recently, MSC-derived EVs, which include both exosomes and microvesicles, are being examined for their therapeutic role in MSC-based cellular therapy since these vesicles are biological entities and are not associated with potential risk factors. In this context, EV-based cell-free therapies are considered promising tools, which improve patients' outcomes considerably with reduced complications in comparison to cell-based therapies (Lai et al., 2013; Fatima and Nawaz, 2015; Armstrong et al., 2017; Toh et al., 2017). Since MSC-derived EVs are non-immunological and the intriguing advantages of stem cell-derived EVs in therapy are largely due to the ability of EVs to stimulate endogenous repair processes within the injured tissue as well as their efficient regulation of immune tolerance and inflammation resolve. Furthermore, EVs could be reproduced in large quantities, easier to handle, are stable off shelf treatment material, less expensive, and do not raise potential ethical and legal issues. However, steering traditional stem cell-based therapies toward EV-based therapies need advance research and rigorous validation *in vitro* and *in vivo* models and in clinical trials. It could be of great interest to applying combination of EV-based therapies with existing approaches in order to improve the therapeutic benefits. Due to their therapeutic potentials, there is a natural desire to test MSC-derived EVs in many diverse clinical indications and there have been proposed best practice to use EVs as therapeutics (Fais et al., 2016; Reiner et al., 2017).

In addition to considering the regulatory effects and functional readouts of EVs, it is important also to consider the type of donor cells and implementation of more sensitive methods for obtaining EVs. To the extent that MSC-derived EVs can be used for cell-free regenerative medicine, much will depend on the quality, reproducibility, and potency of their production, in the same manner that these parameters dictate the development of cell-based MSC therapies (Phinney and Pittenger, 2017). Therefore, a development of highly sensitive platforms and standard operating procedures (SOPs) for obtaining the Good Manufacturing Practice (GMP) grade EVs, and developing best practice in animal models are highly recommended (Fais et al., 2016; Gimona et al., 2017; Reiner et al., 2017).

## Author contributions

FF, KE, IN, MM, HV, AH, GC, and MN participated in writing, reviewing and critical analysis of the manuscript. MN prepared the illustrations, and coordinated the manuscript. All authors agreed and approved the final version.

### Conflict of interest statement

The authors declare that the research was conducted in the absence of any commercial or financial relationships that could be construed as a potential conflict of interest.
